# Alcohol Binge Drinking and Executive Functioning during Adolescent Brain Development

**DOI:** 10.3389/fpsyg.2017.01638

**Published:** 2017-10-04

**Authors:** Soledad Gil-Hernandez, Patricia Mateos, Claudia Porras, Raquel Garcia-Gomez, Enrique Navarro, Luis M. Garcia-Moreno

**Affiliations:** ^1^Department of Didactics and School Organization, Faculty of Education, Complutense University of Madrid, Madrid, Spain; ^2^Department of Psychobiology, Faculty of Education, Complutense University of Madrid, Madrid, Spain; ^3^Department of Methodology, Research, and Diagnosis in Education, Faculty of Education, Complutense University of Madrid, Madrid, Spain

**Keywords:** adolescence, alcohol, binge drinking, executive functioning, history of consumption, prefrontal cortex

## Abstract

Alcohol consumption in adolescents causes negative effects on familiar, social, academic life, as well as neurocognitive alterations. The binge drinking (BD) pattern of alcohol is characterized by the alternation of episodes of heavy drinking in a short interval of time, and periods of abstinence, a practice that can result in important brain alterations; even more than regular alcohol consumption. The prefrontal cortex, which acts as neural support for the executive processes, is particularly affected by alcohol; however, not all studies are in agreement about how BD alcohol consumption affects executive functioning. Some research has found that alcohol consumption in adolescence does not significantly affect executive functioning while others found it does. It is possible that these discrepancies could be due to the history of alcohol consumption, that is, at what age the subjects started drinking. The aim of our study is to assess the performance on executive functioning tasks of 13–19-year-old adolescents according to their pattern of alcohol consumption. We hypothesize that BD adolescents will perform worse than non-BD subjects in tasks that evaluate executive functions, and these differences will increase depending on how long they have been consuming alcohol. Three hundred and twenty-two students (48.14% females; age range 13–22 years; mean aged 16.7 ± 2.59) participated in the study; all of them had begun drinking at the age of 13 years. Participant were divided into three groups, according to their age range (13–15, 16–18, and 19–22 years) and divided according to their pattern of alcohol consumption (BD and control groups). Then, the subjects were evaluated with neuropsychological tasks that assess executive functions like working memory, inhibition, cognitive flexibility, or self-control among others. The entire sample showed a normal improvement in their executive performance, but this improvement was more stable and robust in the control group. Regarding the executive performance among age groups, control subjects only obtained better results than BDs in the 19–22-year-old range, whereas the performance was quite similar at younger ages. Considering that all the BD subjects started drinking at the same age (13 years old), it is possible that a kind of compensation mechanism exists in the adolescent brain which allows them to reach a normal performance in executive tasks. This theoretical mechanism would depend upon neuronal labor, which could lose efficacy over time with further alcohol ingestion. This process would account for the differences in neuropsychological performance, which were only observed in older students with a longer history of alcohol consumption.

## Introduction

Alcohol consumption in adolescents causes negative effects on familiar, social, and academic life, as well as neurocognitive alterations (Jennison, 2004; Jacobus and Tapert, 2013; White and Hingson, 2014). The binge drinking (BD) pattern of alcohol consumption, widespread among adolescents, is characterized by the alternation of episodes of heavy drinking in a short interval of time, and periods of abstinence (Courtney and Polich, 2009). The National Institute on Alcohol Abuse and Alcoholism (NIAAA) has defined “BD” as a pattern of drinking alcohol that brings blood alcohol concentration (BAC) to about 0.08% or above in about 2 h. This pattern corresponds to consuming five or more drinks (male) or four or more drinks (female) in a session at least once in the previous 15–30 days (Courtney and Polich, 2009). BD is responsible for many of the social- and health-related problems affecting adolescents today (Miller et al., 2007; Nelson et al., 2009; Popovici and French, 2013; Kivimaki et al., 2014; Moure-Rodríguez et al., 2014). It is not clear yet if the BD pattern of alcohol consumption can cause brain damage or, by contrast, certain brain abnormalities lead to alcohol abuse (see Petit et al., 2014).

Adolescence is a critical developmental period where some neuromaturational changes lead to significant improvements in complex cognitive functions such as planning, problem solving, working memory, or inhibitory control, namely the executive functions (Luna et al., 2010; Diamond, 2013; Rubia, 2013); however, this maturation process makes these circuits highly vulnerable to the neurotoxic effects of alcohol (Oscar-Berman and Marinkovic, 2007; Bava and Tapert, 2010). The BD pattern of alcohol intake is characterized by repeated episodes of heavy drinking which lead to a great elevation of blood alcohol levels, followed by periods of moderate or null consumption, a practice that can lead to even more important brain alterations than regular alcohol intake (Duka et al., 2003, 2004; Lacaille et al., 2015). The brain transformations seen during adolescence are region-specific and the prefrontal cortex is one of those which mature later (Crews et al., 2007; Casey et al., 2008); this region, that acts as neural support for the executive processes (Fuster, 2001), seems to be particularly affected by alcohol (Weissenborn and Duka, 2003; Hartley et al., 2004; Goudriaan et al., 2007; Scaife and Duka, 2009; Pleil et al., 2015; Trantham-Davidson and Chandler, 2015). This problem could be exacerbated since adolescents are less sensitive than adults to the aversive effects of ethanol, such as the motor impairing, anxiolytic effects, and to hangover discomfort (Spear, 2014); then, they can consume more alcohol before they feel the aversive effects.

There is much scientific literature about the negative effects in neurocognitive performance produced by alcohol consumption, a practice that in adolescence causes a wide variety of neurocognitive deficits with implications for learning and intellectual development (Zeigler et al., 2005). Several studies have revealed the BD’s effects in different cognitive processes, especially in visuospatial abilities, attention, memory, or executive functions (Hartley et al., 2004; Goudriaan et al., 2007; García-Moreno et al., 2008; Johnson et al., 2008; Heffernan et al., 2010; Hanson et al., 2011; Parada et al., 2011a, 2012; Mota et al., 2013; Gil-Hernandez and Garcia-Moreno, 2016; Jones et al., 2016). However, the results of these studies are not fully congruent, especially when executive functions are evaluated.

Executive functions are responsible for control and organize the intentional behavior and are necessary to achieving an adequate adaptation in the society, the school, and the workplace (Jurado and Rosselli, 2007); furthermore, executive functioning develops specifically during adolescence (Crone, 2009) according to the maturation of the parietal and prefrontal cortices (Blakemore and Choudhury, 2006). As we have mentioned before, this prolonged maturational trajectory could explain a particular vulnerability of executive functioning to the effects of alcohol. However, not all studies are in agreement about how BD alcohol consumption affects these processes. For example, some of them have found deficits especially in attention and working memory (Weissenborn and Duka, 2003; Hartley et al., 2004; Townshend and Duka, 2005; García-Moreno et al., 2008; Scaife and Duka, 2009; Sanhueza et al., 2011; Parada et al., 2012), others in decision making (Goudriaan et al., 2007; Johnson et al., 2008), or in tasks of behavioral inhibition (McCarthy et al., 2012; Stautz and Cooper, 2013), planning ability (Weissenborn and Duka, 2003; Hartley et al., 2004; Sanhueza et al., 2011), and cognitive flexibility (Townshend and Duka, 2005; Scaife and Duka, 2009; Sanhueza et al., 2011). However, Gil-Hernandez and Garcia-Moreno (2016) found that adolescents BD scored higher in dysexecutive symptomathology but obtain similar results as the control group in tasks of executive performance. Some authors even argue that heavy drinking does not result in measurable impairments in basic executive functions like sustained attention, inhibition, shift attention, and working memory (Tapert and Brown, 1999; Randall et al., 2004; Landa et al., 2006; Martínez and Manoiloff, 2010; Boelema et al., 2015).

One likely explanation for this variability could be the differences observed in selected samples (age, gender, ethnicity, etc.), the tests used for assessment, or the criteria for calculating alcohol intake (Parada et al., 2011b). Randall et al. (2004) found differences in personality traits both in drinking and non-drinking adolescents and they suggest that differences in personality could be one factor to explain the differences in cognitive performance. They stated that non-drinkers responded to the stress of cognitive testing with a more adverse mood than BD subjects; then, the effects of BD alcohol consumption on neuropsychological performance could be comparable to the effects of stress on the performance of non-drinkers. This is in line with research that supports the idea that moderate alcohol consumption can have health benefits for cognitive functioning (Peele and Brodsky, 2000). The history of alcohol intake, the time since an individual started to drink according to BD pattern, could also help to explain the differences found in this topic; we believe that it is reasonable assumption to think that subjects who have been drinking for longer periods of time exhibit greater neuropsychological alterations than new or recent drinkers. A few years of BD pattern of alcohol consumption may not be enough to damage prefrontal circuits in a sufficient level to exhibit cognitive deterioration. However, acute alcohol intake causes early brain alterations (Spagnolli et al., 2013; Zheng, 2017), ergo some kind of compensatory mechanism must have been implemented, by which BD subjects obtained similar scores to non-drinkers. This compensatory mechanism would depend upon neuronal effort, which could lose efficiency over time if alcohol ingestion doesn’t stop.

The aim of our study is to assess the effect of history of alcohol consumption on the performance in executive functioning tasks in a sample of 13–20 years old adolescents who had begun to drink at 13 years old. We hypothesize that BD adolescents will obtain worse results than non-BD subjects in test of executive functions, such as working memory, cognitive flexibility, or self-control, among others. Moreover, it has been hypothesized that the older adolescents will exhibit higher differences since they will have been drinking for a longer period of time.

## Materials and Methods

This study is part of a broader project, which we have been conducting for the last several years. The material and procedures have been previously described (Gil-Hernandez and Garcia-Moreno, 2016); here, we will outline some of them.

### Participants

Three hundred and twenty-two students (age range 13–22 years; mean aged 16.7 ± 2.59) participated in the study; 48.14% women (*n* = 155, mean aged 16.97 ± 2.67) and 51.86% men (*n* = 167, mean aged 16.44 ± 2.5). First, all participants, who were students from secondary schools and universities in Madrid (Spain), fulfilled a self-referred questionnaire (ESAJ-S) collectively in their classrooms. This questionnaire was developed specifically for these studies and includes questions about demographic, medical, social, and personal features of the subject, the full version of the Alcohol Use Disorders Identification Test (AUDIT, Saunders et al., 1993), and questions related to the use of alcohol (number of BD episodes, age of onset on alcohol consumption, etc.). In order to evaluate the items related to alcohol consumption we took the recommendations of the World Health Organization (2000) and the specifications of the European School Survey Project: Alcohol and other Drugs (The European School Survey Project on Alcohol and Other Drugs [ESPAD], 2011) into account. The sample utilized in the study was obtained from the group of participants in the wider research; the inclusion criterion was having started drinking following a BD pattern before they turned 14 years old. Regular consumption of cannabis or other drugs, personal history of neurological or relevant systemic disease, personal or familiar alcohol use disorder (DSM-IV criteria), major mental disorder, and history of alcoholism in first-degree relatives were considered as exclusion criteria. Smokers and sporadic cannabis users (two joints or less in a month) were not excluded from the study.

The students selected were assigned to one of three groups according to their age (13–15 years old, *n* = 112; 16–18 years old, *n* = 109; and 19–22 years old, *n* = 101). Then, within each of those groups, the subjects were assigned to one of two groups according to their pattern of alcohol consumption (**Table 1**). The country in which the study is being carried out must be taken into account, because there are differences in the grams of alcohol of the Standard Drinking Units (SDUs) among countries. For instance, a SDU in the United States contains 14 g of ethanol, 8 g in the United Kingdom, and 10 g in Spain. To avoid these variations we used the criterion of the World Health Organization (2000) and the groups were as follows:

- Binge drinking (intensive alcohol consumption): Subjects who drink more than 6 (men) or 4 (women) SDU (10 g each) during one episode of intake (3–4 continuous hours) at least once a month in the last 6 months. All subjects from this group had begun to drink at the age of 13 years, that is, they had experienced a BD episode before the age of 14 years and then continued to drink.
- CTR (control group): Subjects who do not consume any alcohol or only do it on special occasions (birthdays, new year, etc.).

**Table 1:** Configuration of the experimental groups and consumption features.

	13–15 (*n* = 112)	16–18 (*n* = 109)	19–22 (*n* = 101)
BD	Age: 13.82 ± 0.93	Age: 17.13 ± 0.83	Age: 19.75 ± 0.88
	♀: *n* = 16 (13.75 ± 1)	♀: *n* = 28 (17.04 ± 0.82)	♀: *n* = 36 (19.48 ± 0.51)
	♂: *n* = 22 (13.86 ± 0.89)	♂: *n* = 33 (17.21 ± 0.82)	♂: *n* = 23 (19.9 ± 0.85)
	Audit total^∗^**^,+^**: 11.72 ± 2.3	Audit total^∗^: 13.16 ± 2.1	Audit total^∗^: 12.27 ± 1.79
	BDE-3m^∗^**^,+^**: 2.66 ± 0.94	BDE-3m^∗^: 4.51 ± 1.23	BDE-3m^∗^: 3.42 ± 0.72
	Tobacco^∗∗^: 31.6%	Tobacco^∗∗^: 44.3%	Tobacco^∗^: 54.2%
	Cannabis: 7.9%	Cannabis: 14.8%	Cannabis: 16.9%
CTR	Age: 13.66 ± 0.8	Age: 16.75 ± 0.81	Age: 19.67 ± 0.93
	♀: *n* = 33 (13.70 ± 0.81)	♀: *n* = 19 (16.68 ± 0.82)	♀: *n* = 23 (19.92 ± 1.02)
	♂: *n* = 41 (13.63 ± 0.8)	♂: *n* = 29 (16.79 ± 0.84)	♂: *n* = 19 (19.43 ± 0.95)
	Audit total^∗^: 1.28 ± 1.09	Audit total^∗^: 2.27 ± 1.14	Audit total^∗^: 2.81 ± 0.94
	BDE-3m^∗^: 0	BDE-3m^∗^: 0	BDE-3m^∗^: 0
	Tobacco^∗∗^: 10.8%	Tobacco^∗∗^: 16.7%	Tobacco^∗^: 33.3%
	Cannabis: 1.4%	Cannabis: 6.2%	Cannabis: 7.1%


*BDE-3m: binge drinking (BD) episodes experienced by subjects in the last 3 months. ^∗^*p* < 0.01; ^∗∗^*p* < 0.05 between BD and CTR groups. ^+^*p* < 0.01 BD group scores in the three age groups. The *post hoc* analysis shows significant differences in AUDIT and BDE between 13–15 years and the others age groups, but there is no difference between 16–18 and 19–22 years.*

According to the original procedure (Gil-Hernandez and Garcia-Moreno, 2016), neuropsychological assessments were conducted between Tuesday and Thursday to avoid the proximity of the weekend, and participants were asked to abstain from consuming drugs and alcohol within 24 h prior to tests. The testing took place individually in the University and participating centers’ premises. Students voluntarily participated in the study after being fully informed of the objectives and process of the study. In all cases, including those who were over 18 years, the parents were informed and they signed a consent form. The study was exempt from ethical approval procedures; however, all procedures are in accordance with the Spanish legislation, Law 14/2007 of July 3, the Code of Ethical Principles for Medical Research Involving Humans Subjects outlined in the Declaration of Helsinki, and the Ethical Principles of Psychologists and Code of Conduct according to the American Psychological Association.

### Materials and Measures

The subjects were evaluated with the following neuropsychological tools:

#### Subtests of the Wechsler Memory Scale (WMS-III; Wechsler, 1997)

- Digits and spatial span (forward and backward condition). These tests are commonly used to evaluate short-term verbal and spatial memory (Richardson, 2007) as well the executive component of these cognitive processes (Baddeley, 2003). In the digits test, the subjects have to repeat sequences of numbers of increasing difficulty in direct or reverse order (working memory); and the number of successful sequences was recorded (DIG-F and DIG-B). The spatial span has a similar procedure, but the subjects have to repeat the sequence in which the examiner taps cubes placed on a board, in direct or reverse order; the number of successful sequences was recorded too (SS-F and SS-B).
- Letter–Number sequencing subtest. The subject is presented with a mixed list of numbers and letters and their task is to repeat the list by saying the numbers first in ascending order and then the letters in alphabetical order. This subtest appears to require more than just immediate memory and there is no minimum academic skill prerequisite other than knowing the numbers 1–9 and having a functional knowledge of the alphabet. Moreover, the Letter–Number Sequencing subtest has high face validity as a working memory task (Hill et al., 2010). The number of successful sequences was recorded (LN).

#### Verbal Fluency

Verbal fluency is a cognitive function that facilitates information retrieval from memory. Tests of verbal fluency evaluate an individual’s ability to retrieve specific information within restricted search parameters (Lezak et al., 2004). Successful retrieval depends upon executive control over cognitive processes such as selective attention, selective inhibition, mental set shifting, internal response generation, and self-monitoring. Verbal fluency tasks have shown to produce brain activation in the prefrontal dorsolateral region of the left hemisphere (Gourovitch et al., 2000). Prefrontal activation during phonemic and semantic verbal fluency tasks is higher than the one observed in other verbal task, where generating and self-monitoring items is not necessary (specific words, in this case) (Kono et al., 2007; Tupak et al., 2012). The task included two conditions:

- Phonemic fluency: The participants had to say loudly as many words as possible that begin with the letter F in 1 min, then with A, and finally, with S. The number of total correct words was recorded (PhF).
- Semantic fluency: The participants had to say loudly as many names as possible of animals (1 min) and fruits (1 min). The number of total correct words was recorded (SF).

#### Trail Making Test (TMT; Reitan, 1992)

The trail making test (TMT) is a neuropsychological tool commonly used to assess executive processes such as attention, cognitive flexibility, working memory, and other executive functions (Lezak et al., 2004; Mitrushina et al., 2005; Strauss et al., 2006). In TMT-A, the participant must draw a line connecting a series of numbers in sequential order. In TMT-B, the subjects have to carry out the same processes, but including letters in alphabetical order. The time spent on completing both parts (TMT-A and TMT-B), and the difference between time B and time A (TMT-BA) is recorded.

#### Stroop Color–Word Task (Stroop, 1935)

This well-know test is an appropriate procedure for examining selective attention and cognitive flexibility. This task can be applied following several different formats; we chose Golden’s (1978) method, which consist of three pages with (i) color words printed in black ink, (ii) color hues printed as XXXX, and (iii) color hues printed as competing color words (e.g., “green” printed in red ink), respectively. The participants had 45 s to read correctly each page. The variables recorded in this task were the number of words read (STP-W), the number of colors named (STP-C), and the items with word–color interference (STP-WC). Interference is caused by a color word printed in an incongruent color, leading to slower reactions and more errors as compared to color words printed in the congruent color and neutral words not printed in a color; an interference index was calculated too (STP-I). Recent neuroimaging studies have shown that especially the rostral cingulate zone and the dorsolateral prefrontal cortex become active during interference (Ridderinkhof et al., 2004; Carter and van Veen, 2007).

### Statistical Analysis

First, we determined the normality of variables’ distribution by the Kolmogorov–Smirnov test, and used the Levene’s test to prove the homoscedasticity between BD and control groups. We have used Student’s *t*-test to analyze the group mean differences in Audit and BDE-3m variables, and the Chi-square test for the frequency differences in tobacco and cannabis consumption. Then, we use an ANOVA to compare Audit scores and BDE-3m from BD subjects in the three age groups. To prove the natural age-related improvement in executive functioning, we carried out Pearson’s correlation analyses between the age of the subjects and their performance in executive tasks for the whole sample and for both groups separately; then, we calculate a Fisher *r*-to-*z* transformation to test differences between correlation coefficients. After this, we used Student’s *t*-test to check the possible average differences between control and BD groups, studying each age group separately. Finally, we calculate Cohens’ *d* effect size (Cohen, 1988) to test the magnitude of the difference. Differences were considered statistically significant at *p* < 0.05. The data were analyzed by use of the IBM SPSS statistics package for Windows, version 23.0.

## Results

We analyzed the descriptive features of the sample (**Table 1**). The BD and CTR groups exhibited differences in the total score of the Audit test in the three age groups {13–15: [*t*(45.75) = -26.45; *p* = 0.000]; 16–18: [*t*(96.31) = -34.56; *p* = 0.000]; and 19–22: [*t*(92.24) = -34.45; *p* = 0.000]}, where the BD subject scored higher than CTR ones. We also found significant differences in the DBE-3m values {13–15: [*t*(37) = -17.47; *p* = 0.000]; 16–18: [*t*(60) = -28.55; *p* = 0.000]; and 19–22: [*t*(58) = -36.32; *p* = 0.000]}, where again the BD subject scored higher than CTR ones. In relation with tobacco consumption, the percentage of smokers in the BD group was significantly higher than in CTR in the three age groups (13–15: χ^2^ = 7.38, *p* = 0.007; 16–18: χ^2^ = 9.38, *p* = 0.007; and 19–22: χ^2^ = 7.38, *p* = 0.038). However, no differences were found in cannabis use (13–15: χ^2^ = 3.12, *p* = 0.08; 16–18: χ^2^ = 1.98, *p* = 0.16; and 19–22: χ^2^ = 2.15, *p* = 0.15). When we compared BD subjects from the three age groups, we found significant differences in Audit total scores (*F* = 16.24, *p* = 0.000) and in BDE-3m episodes (*F* = 20.72, *p* = 0.000). The *post hoc* analysis revealed that the 13–15 group scored significantly lower than the 16–18 and 19–22 groups in both variables, and that no differences were found between these two groups.

**Table 2** shows the Pearson correlation indexes found between executive variables and age of the subjects. As expected, when we study the whole sample, all neuropsychological variables correlate significantly with the age of the subjects, indicating that executive functioning improves with age in all subjects, irrespective of experimental group to which they belong. Negative values of the *r* in TMT variables indicate a negative correlation because these scores reflect the time spent solving the task and a higher time indicates a worse performance. Nevertheless, there were differences in the correlation coefficients between BD and CTR groups; specifically, these significant differences were observed in SS-F, LN, TMT, and STP-C and STP-WC. In all cases, the direction of correlation was equal but changes the value except in the STP-WC variable, where both values are close to zero.

**Table 2:** Correlations between age of the subjects and the scores in the executive tasks, first in the whole sample and after, separated by groups.

		All		CTR		BD		CTR vs. BD	
					
Test	Variable	*r*	Sig.	*r*	Sig.	*r*	Sig.	Fisher *Z*	*p*
Digits (WMS-III)	DIG-F	0.125	**0.012**	0.164	**0.036**	0.04	0.615	0.95	0.342
	DIG-B	0.097	**0.042**	0.087	0.266	0.156	**0.050**	-0.62	0.535
Spatial span (WMS-III)	SS-F	0.297	**0.000**	0.386	**0.000**	0.182	**0.022**	1.98	**0.047**
	SS-B	0.471	**0.000**	0.502	**0.000**	0.402	**0.000**	1.12	0.263
Letter–number (WMS-III)	LN	0.270	**0.000**	0.375	**0.000**	0.167	**0.036**	2.01	**0.044**
Verbal fluency	PHF	0.273	**0.000**	0.270	**0.000**	0.123	0.125	1.36	0.174
	SF	0.185	**0.000**	0.183	**0.019**	0.088	0.272	0.86	0.390
Trail making test	TMT-A	-0.223	**0.000**	-0.406	**0.000**	-0.018	0.826	-3.67	**0.000**
	TMT-B	-0.326	**0.000**	-0.507	**0.000**	-0.147	0.065	-3.65	**0.000**
	TMT-BA	-0.265	**0.000**	-0.397	**0.000**	-0.153	0.055	-2.36	**0.020**
Stroop test	STP-W	0.383	**0.000**	0.447	**0.000**	0.286	**0.000**	1.66	0.100
	STP-C	0.370	**0.000**	0.500	**0.000**	0.252	**0.001**	2.59	**0.010**
	STP-WC	0.144	**0.005**	0.181	**0.020**	-0.011	0.891	1.72	**0.090**
	STP-I	-0.143	**0.005**	-0.217	**0.005**	-0.234	**0.003**	0.16	0.873


*Significant values are in bold.*

With Student’s *t*-test we didn’t find statistical differences between BD and CTR groups at 13–15 age range (**Table 3**). Something similar occurs with subjects 16–18 years old; in this case, we only found differences in the *digits forward* test (*t-* and *p-*values are provided in the corresponding table), where the BD group obtained better results than the CTR group (**Table 4**) with a moderate effect size (*d* = 0.518); it means that BD group performs roughly 0.5 standard deviations above CTR group. Subjects between 19 and 22 years of age exhibited more performance differences according to their alcohol consumption pattern (**Table 5**). The CTR group performed better in *SS-F* with a low to moderate effect size (*d* = 0.468), in *LN* with a moderate to high difference (*d* = 0.705), in the three variables of the *TMT* (*TMT-A, TMT-B*, and *TMT-BA*) with a high effect size in the first and second case (*d* = 1.473 and *d* = 0.955, respectively) and low to moderate in the third one (*d* = 0.449), and in *STP-C* task with a moderate to high difference (*d* = 0.755). As we stated before, negative values of the *t* in TMT variables indicate higher scores of BD subjects, that is, a worse performance of this group.

**Table 3:** Mean differences between BD and control groups of 13–15-year-old subjects.

		13–15 years group						
			
Test	Variable	Group	Mean	*SD*	*t*	*DF*	Sig.	Cohen’s *d*
Digits (WMS-III)	DIG-F	CTR	7.32	1.84	-0.048	110	0.962	
		BD	7.34	1.88				
	DIG-B	CTR	6.73	1.54	1.215	110	0.227	
		BD	6.34	1.72				
Spatial span (WMS-III)	SS-F	CTR	7.14	1.62	0.696	110	0.488	
		BD	6.92	1.36				
	SS-B	CTR	6.26	1.73	0.452	110	0.652	
		BD	6.11	1.57				
Letter–number (WMS-III)	LN	CTR	10.14	2.17	0.073	110	0.942	
		BD	10.11	1.78				
Verbal fluency	PHF	CTR	37.77	8.26	0.321	110	0.749	
		BD	37.26	7.21				
	SF	CTR	31.28	6.78	0.532	110	0.596	
		BD	30.58	6.36				
Trail making test	TMT-A	CTR	31.04	8.89	-0.452	110	0.652	
		BD	31.87	9.70				
	TMT-B	CTR	72.50	18.16	-0.325	110	0.746	
		BD	73.66	17.27				
	TMT-BA	CTR	41.46	15.49	-0.110	110	0.913	
		BD	41.79	14.15				
Stroop test	STP-W	CTR	98.49	12.92	-0.016	110	0.988	
		BD	98.53	12.62				
	STP-C	CTR	68.92	10.44	0.089	110	0.930	
		BD	68.74	10.01				
	STP-WC	CTR	45.57	10.79	-0.692	110	0.491	
		BD	46.97	8.88				
	STP-I	CTR	5.13	7.18	-1.111	110	0.269	
		BD	6.64	6.02				


**Table 4:** Mean differences between BD and control groups of 16–18-year-old subjects.

		16–18 years group						
			
Test	Variable	Group	Mean	*SD*	*t*	*DF*	Sig.	Cohen’s *d*
Digits (WMS-III)	DIG-F	CTR	7.04	1.41	-2.681	107	**0.009**	**0.518**
		BD	7.79	1.46				
	DIG-B	CTR	6.33	1.73	-1.612	107	0.110	
		BD	6.82	1.42				
Spatial span (WMS-III)	SS-F	CTR	7.85	1.52	-1.061	107	0.291	
		BD	8.19	1.79				
	SS-B	CTR	6.65	1.66	-1.6	107	0.112	
		BD	7.21	1.97				
Letter–number (WMS-III)	LN	CTR	11.25	1.91	0.29	107	0.772	
		BD	11.15	1.77				
Verbal fluency	PHF	CTR	41.17	8.39	-0.555	107	0.580	
		BD	42.11	9.19				
	SF	CTR	32.96	6.46	-1.189	107	0.237	
		BD	34.51	6.98				
Trail making test	TMT-A	CTR	28.06	7.08	-0.714	107	0.477	
		BD	29.07	7.44				
	TMT-B	CTR	62.33	14.90	-0.116	106.93	0.908	
		BD	62.71	18.49				
	TMT-BA	CTR	34.27	14.02	0.205	107	0.838	
		BD	33.64	17.33				
Stroop test	STP-W	CTR	113.04	12.75	0.888	107	0.377	
		BD	110.89	12.45				
	STP-C	CTR	79.77	12.16	1.52	107	0.132	
		BD	76.31	11.51				
	STP-WC	CTR	52.02	8.67	0.068	107	0.946	
		BD	51.90	9.47				
	STP-I	CTR	5.43	6.68	-1.156	107	0.250	
		BD	6.91	6.59				


*Significant values are in bold.*

**Table 5:** Mean differences between BD and control groups of 19–22-year-old subjects.

		19–22 years group						
			
Test	Variable	Group	Mean	*SD*	*t*	*DF*	Sig.	Cohen’s *d*
Digits (WMS-III)	DIG-F	CTR	8.02	1.47	1.359	97.448	0.177	
		BD	7.58	1.83				
	DIG-B	CTR	7.19	1.71	0.575	99	0.567	
		BD	7.00	1.59				
Spatial span (WMS-III)	SS-F	CTR	8.88	1.99	2.309	99	**0.023**	**0.468**
		BD	7.93	2.07				
	SS-B	CTR	8.93	1.83	1.299	99	0.197	
		BD	8.37	2.30				
Letter–number (WMS-III)	LN	CTR	12.02	1.57	3.509	99	**0.001**	**0.705**
		BD	10.95	1.48				
Verbal fluency	PHF	CTR	42.93	6.06	1.925	99	0.057	
		BD	40.39	6.85				
	SF	CTR	34.14	5.76	1.329	99	0.187	
		BD	32.58	5.89				
Trail making test	TMT-A	CTR	22.64	5.24	-7.193	99	**0.000**	**1.473**
		BD	31.10	6.21				
	TMT-B	CTR	49.86	11.41	-4.801	**94.346**	**0.000**	**0.995**
		BD	65.17	20.43				
	TMT-BA	CTR	27.21	8.75	-2.386	**85.806**	**0.019**	**0.449**
		BD	34.07	19.47				
Stroop test	STP-W	CTR	113.98	14.97	1.473	99	0.144	
		BD	109.81	13.27				
	STP-C	CTR	83.00	7.71	3.678	99	**0.000**	**0.755**
		BD	76.46	9.51				
	STP-WC	CTR	49.09	7.70	0.915	99	0.362	
		BD	47.51	9.16				
	STP-I	CTR	0.57	8.98	-1.109	99	0.270	
		BD	2.52	8.48				


*Significant values are in bold.*

## Discussion

The main objective of the present study was to determine the effects of the history of BD alcohol consumption on executive functioning during adolescent brain development in students who started to drink at the age of 13 years. Firstly, our results show that both BD drinkers and non-drinkers progressively improve their executive functioning with age; however, CTR subjects showed a clear age-related improvement whereas BD subjects do not. Executive functions emerge early in child development and change significantly during the preschool years, but they continue to develop during adolescence in parallel with the development of the prefrontal cortex (Zelazo et al., 2008). With increasing age, prefrontal activity becomes more focal and specialized while irrelevant and diffuse activity in this region is reduced (Brown et al., 2005; Durston et al., 2006). During adolescent development an improvement in intellectual functioning occurs in certain number of functions like speed of processing, sustained attention, abstract thought, working memory, set shifting, decision making and planning, and response inhibition (Rubia et al., 2000, Bedard et al., 2002; Rueda et al., 2004; Blakemore and Choudhury, 2006; Crone et al., 2006a,b; Casey et al., 2008; Yurgelun-Todd, 2007; Geier et al., 2009, 2010). BD alcohol consumption affects prefrontal cortex development and could interfere with the normal improvement of neurocognitive abilities like executive functions (Parada et al., 2012). In general, our results are consistent with these findings; however, something different occurs when we compare the level of improvement in BD and control subjects.

We found no differences in executive performance between CTR and BD subjects from 13 to 18 years of age; however, the 19–22 years BD subjects obtained worse scores in several executive tasks. Correlation between alcohol consumption and other drugs in adolescence and structural and functional alterations in different brain regions has already been documented (see Feldstein et al., 2014) as well as a decline in performance on neuropsychological tests of attention, memory, or executive functions (see Dager et al., 2013). However, in our study, BD adolescents until the age of 18 years have shown similar performance to that of the controls, and even better in some tests; with these results we cannot state that the BD pattern has affected executive functioning at this age. It seems BD has no impact on the neuropsychological performance of adolescents with no more than 5 years of alcohol consumption, that is to say, a short history of alcohol consumption. A possible cause could be the characteristics of the tests used in this study. Many of the tests used to assess executive functioning come from clinical settings and were originally designed to measure other psychological processes (Lezak et al., 2004). For this reason, these tests are very useful when are used in people with a high degree of brain deterioration, but they can be less accurate when are used in healthy subjects. In our study, we assessed healthy adolescent students, with a short history of alcohol consumption and without problems in their familiar, social, and academic life. In order to determine the early effects of the BD, Barkley (2011) proposed an alternative procedure, the use of scales of executive functioning or the observation of subjects’ performance in daily activities. In a sample of 12–18-year-old students, Gil-Hernandez and Garcia-Moreno (2016) found no differences between BD and control subjects on executive performance tasks, but the BD group exhibited a more pronounced dysexecutive symptomatology with problems related to inhibition, intentionality, or executive memory. Then, a possible explanation for the absence of differences on executive functioning could be a limited capacity of the test used to discriminate the effects of prefrontal deterioration in these subjects. However, these same tests are capable of finding differences in executive performance between BD and control subjects when the 19–22-year-old groups are assessed. Then, it looks like regular alcohol consumption would progressively damage neuronal circuits until a point when cognitive failure would be evident. Differences in results between executive performance tests and dysexecutive questionnaires can reveal a latent dysfunction in prefrontal circuits whose effects are not evident in neuropsychological tasks but affect daily activities. Concerning this, we want to point out to several studies that have already established that a moderate dose of alcohol is sufficient to affect inhibitory control (see a review in Field et al., 2010), yet not enough to produce evident alterations in other neuropsychological tests.

An alternative explanation may be the existence of a different pattern of brain activation to solve the same task. Crego et al. (2010) found no significant differences between the control and BD groups in a working memory task; however, with event-related potentials, they found a hypoactivation in the anterior prefrontal cortex of BD subjects compared to control ones during the cognitive task. They argued that this apparent inconsistence between the cognitive and the neurophysiological results could be due to the low sensitivity of the task used or to the short history of alcohol consumption of the subjects from the BD group. Other brain imaging studies have found both decreased and increased brain activity in several brain regions during memory and executive tasks (Schweinsburg et al., 2011; Squeglia et al., 2011; Xiao et al., 2013). Then, the fact that BD adolescents didn’t show worse results than non-drinkers in neuropsychological tests but they exhibited different patterns of brain activation could mean that some kind of compensatory mechanism exists in brain activity of BD subjects which allows them to obtain an adequate performance (Campanella et al., 2013). This means that an additional recruitment of neural resources would be required in BD subjects to perform the tasks with the same level of performance as the control group, something that has been observed with other cognitive processes (Zölliga et al., 2010). A study with verbal memory and fMRI has shown that BD adolescents require the activation of more cerebral areas than CTR subjects to solve these neuropsychological tasks with a similar level of performance (Schweinsburg et al., 2010). Two related studies with even-related potentials founded that BD subjects showed a higher neural activation than control subjects in their EEG records in several neuropsychological tasks where both groups demonstrated similar performance (López-Caneda et al., 2012, 2013). According to the authors, the results may reflect the use of additional neural resources in order to successfully attend the demands of the task and that, when BD alcohol intake stops, this neural recruitment is diminished. Nonetheless, the neuronal effort required could lose efficiency over time if alcohol ingestion doesn’t stop. This loss of efficiency can be the explanation for the worst neuropsychological performance in older subjects with a longer history of BD alcohol consumption (Hartley et al., 2004; Goudriaan et al., 2007; García-Moreno et al., 2008, 2009; Parada et al., 2011a, 2012; Sanhueza et al., 2011).

In a nutshell, both BD and control subjects develop cognitive and intellectual abilities normally throughout adolescence. However, subjects who drink alcohol heavily show an incipient deterioration of their performance over time; before this, brain circuits exhibit some signs of alteration especially in prefrontal areas. Heavy alcohol drinking in adolescents leads to a certain dysfunction of prefrontal circuits, which only manifest after several years of BD pattern maintenance. Prefrontal dysfunction is not so clearly demonstrated in the neuropsychological tests because BD subjects score negatively only after time has passed with alcohol consumption. It is not absolutely clear whether the prefrontal signs might have been caused by alcohol intake or they were present before the start of alcohol consumption. We are going to abide by the first option since some of these signs can experience some changes if the alcohol intake stops (López-Caneda et al., 2014; Carbia et al., 2017). Nevertheless, more interdisciplinary research is necessary, especially with earlier age groups in order to determine the brain nets configuration before the start of alcohol intake and their changes once the consumption has been began. All in all, we believe there is a need to more thoroughly study the deleterious effects of alcohol consumption in young people to detect early signs of effects on the brain, and to design more effective interventions.

## Author Contributions

LG-M and SG-H have designed the study; SG-H, PM, CP, and RG-G carried out the neuropsychological assessment; LG-M and EN performed data analysis; and all authors have participated in the drafting and revision of the manuscript, and they have approved the final version for publication.

## Conflict of Interest Statement

The authors declare that the research was conducted in the absence of any commercial or financial relationships that could be construed as a potential conflict of interest. The reviewer GP and handling Editor declared their shared affiliation, and the handling Editor states that the process nevertheless met the standards of a fair and objective review.

## References

[B1] Baddeley A. (2003). Working memory: looking back and looking forward. *Nat. Rev.* 4 829–839. 10.1038/nrn1201 14523382

[B2] Barkley R. A. (2011). *Barkley Deficits in Executive Functioning Scale (BDEFS).* New York, NY: The Guilford Press.

[B3] Bava S. Tapert S. F. (2010). Adolescent brain development and the risk for alcohol and other drug problems. *Neuropsychol. Rev.* 20 398–413. 10.1007/s11065-010-9146-6 20953990 PMC2988999

[B4] Bedard A. C. Nichols S. Barbosa J. A. Schachar R. Logan G. D. Tannock R. (2002). The development of selective inhibitory control across the life span. *Dev. Neuropsychol.* 21 93–111. 10.1207/S15326942DN2101-5 12058837

[B5] Blakemore S. Choudhury S. (2006). Development of the adolescent brain: implications for executive function and social cognition. *J. Child Psychol. Psychiatry* 47 296–312. 10.1111/j.1469-7610.2006.01611.x 16492261

[B6] Boelema S. R. Harakeh Z. Van Zandvoort M. J. E. Reijneveld S. A. Verhulst F. C. Ormel J. (2015). Adolescent heavy drinking does not affect maturation of basic executive functioning: longitudinal findings from the TRAILS Study. *PLOS ONE* 10:e0139186 10.1371/journal.pone.0139186 PMC4619383 26489080

[B7] Brown T. T. Lugar H. M. Coalson R. S. Miezin F. M. Petersen S. E. Schlaggar B. L. (2005). Developmental changes in human cerebral functional organization for word generation. *Cereb. Cortex* 5 275–290. 10.1007/s11065-010-9145-7 15297366

[B8] Campanella S. Peigneux P. Petit G. Lallemand F. Saeremans M. Noel X. (2013). Increased cortical activity in binge drinkers during working memory task: a preliminary assessment through a functional magnetic resonance imaging study. *PLOS ONE* 8:e62260 10.1371/journal.pone.0062260 PMC3636085 23638017

[B9] Carbia C. Cadaveira F. Lopez-Caneda E. Caamaño-Isorna F. Rodríguez Holguín S. Corral M. (2017). Working memory over a six-year period in young binge drinkers. *Alcohol* 61 17–23. 10.1016/j.alcohol.2017.01.013 28599713

[B10] Carter C. S. van Veen V. (2007). Anterior cingulate cortex and conflict detection: an update of theory and data. *Cogn. Affect. Behav. Neurosci.* 7 367–379. 10.3758/CABN.7.4.367 18189010

[B11] Casey B. Jones R. M. Hare T. A. (2008). The adolescent brain. *Ann. N. Y. Acad. Sci.* 1124 111–126. 10.1196/annals.1440.010 18400927 PMC2475802

[B12] Cohen J. (1988). *Statistical Power Analysis for the Behavioral Sciences*, 2nd. Edn Hillsdale, NJ: Erlbaum.

[B13] Courtney K. E. Polich J. (2009). Binge drinking in young adults: data, definitions, and determinants. *Psychol. Bull.* 135 142–156. 10.1037/a0014414 19210057 PMC2748736

[B14] Crego A. Rodríguez Holguín S. Parada M. Mota N. Corral M. Cadaveira F. (2010). Reduced anterior prefrontal cortex activation in young binge drinkers during a visual working memory task. *Drug Alcohol Depend.* 109 45–56. 10.1016/j.drugalcdep.2009.11.020 20079980

[B15] Crews F. He J. Hodge C. (2007). Adolescent cortical development: a critical period of vulnerability for addiction. *Pharmacol. Biochem. Behav.* 86 189–199. 10.1016/j.pbb.2006.12.001 17222895 PMC11646682

[B16] Crone E. A. (2009). Executive functions in adolescence: inferences from brain and behavior. *Dev. Sci.* 12 825–830. 10.1111/j.1467-7687.2009.00918.x 19840037

[B17] Crone E. A. Donohue S. E. Honomichl R. Wendelken C. Bunge S. A. (2006a). Brain regions mediating flexible rule use during development. *J. Neurosci.* 26 11239–11247. 10.1523/JNEUROSCI.2165-06.2006 17065463 PMC6674662

[B18] Crone E. A. Wendelken C. Donohue S. van Leijenhorst L. Bunge S. A. (2006b). Neurocognitive development of the ability to manipulate information in working memory. *Proc. Natl. Acad. Sci. U.S.A.* 103 9315–9320. 10.1073/pnas.0510088103 16738055 PMC1472660

[B19] Dager A. Squeglia L. Castro N. Tapert S. F. (2013). “Addiction and the human adolescent brain,” in *Biological Research on Addiction. Comprehensive Addictive Behaviors and Disorders* Vol. 2 ed. Miller P. M. (San Diego, CA: Academic Press), 353–364.

[B20] Diamond A. (2013). Executive functions. *Annu. Rev. Psychol.* 64 135–168. 10.1146/annurev-psych-113011-143750 23020641 PMC4084861

[B21] Duka T. Gentry J. Malcolm R. Ripley T. L. Borlikova G. Stephens D. N. (2004). Consequences of multiple withdrawals from alcohol. *Alcohol. Clin. Exp. Res.* 28 233–246. 10.1186/s12888-016-0757-1 15112931

[B22] Duka T. Townshend J. M. Collier K. Stephens D. N. (2003). Impairment in cognitive functions after multiple detoxifications in alcoholic inpatients. *Alcohol. Clin. Exp. Res.* 27 1563–1572. 10.1097/01.ALC.0000090142.11260.D7 14574226

[B23] Durston S. Davidson M. C. Tottenham N. Galvan A. Spicer J. Fossella J. A. (2006). A shift from diffuse to focal cortical activity with development. *Dev. Sci.* 9 1–8. 10.1111/j.1467-7687.2005.00454.x 16445387

[B24] Feldstein S. W. Sakhardande A. Blakemore S. J. (2014). The effect of alcohol consumption on the adolescent brain: a systematic review of MRI and fMRI studies of alcohol-using youth. *Neuroimage Clin.* 5 420–437. 10.1016/j.nicl.2014.06.011 26958467 PMC4749850

[B25] Field M. Wiers R. V. Christiansen P. Fillmore M. T. Verster J. C. (2010). Acute alcohol effects on inhibitory control and implicit cognition: implications for loss of control over drinking. *Alcohol. Clin. Exp. Res.* 34 1346–1352. 10.1111/j.1530-0277.2010.01218.x 20491732 PMC2999764

[B26] Fuster J. M. (2001). The prefrontal cortex-an update: time is of the essence. *Neuron* 30 319–333. 10.1016/S0896-6273(01)00285-9 11394996

[B27] García-Moreno L. M. Expósito F. J. Sanhueza C. Gil-Hernández S. (2009). Rendimiento neurocognitivo y alcoholismo de fin de semana en adolescentes. *Rev. Psicol. Educ.* 3 163–176.

[B28] García-Moreno L. M. Expósito J. Sanhueza C. Angulo M. T. (2008). Prefrontal activity and weekend alcoholism in the young. *Adicciones* 20 271–280. 10.20882/adicciones.269 18813773

[B29] Geier C. F. Garver K. Terwilliger R. Luna B. (2009). Development of working memory maintenance. *J. Neurophysiol.* 101 84–99. 10.1152/jn.90562.2008 18971297 PMC2637004

[B30] Geier C. F. Terwilliger R. Teslovich T. Velanova K. Luna B. (2010). Immaturities in reward processing and its influence on inhibitory control in adolescence. *Cereb. Cortex* 20 1613–1629. 10.1093/cercor/bhp225 19875675 PMC2882823

[B31] Gil-Hernandez S. Garcia-Moreno L. M. (2016). Executive performance and dysexecutive symptoms in binge drinking adolescents. *Alcohol* 51 79–87. 10.1016/j.alcohol.2016.01.003 26992704

[B32] Golden C. J. (1978). *Stroop Color and Word Test.* Wood Dale, IL: Stoelting Co.

[B33] Goudriaan A. E. Grekin E. R. Sher K. J. (2007). Decision making and binge drinking: a longitudinal study. *Alcohol. Clin. Exp. Res.* 31 928–938. 10.1111/j.1530-0277.2007.00378.x 17403069 PMC2667377

[B34] Gourovitch M. L. Kirkby B. S. Goldberg T. E. Weinberger D. R. Gold J. M. Esposito G. (2000). A comparison of rCBF patterns during letter and semantic fluency. *Neuropsychology* 14 353–360. 10.1037/0894-4105.14.3.353 10928738

[B35] Hanson K. L. Medina K. L. Padula C. B. Tapert S. F. Brown S. A. (2011). Impact of adolescent alcohol and drug use on neuropsychological functioning in young adulthood: 10-year outcomes. *J. Child. Adolesc. Subst. Abuse* 20 135–154. 10.1080/1067828X.2011.555272 21532924 PMC3083020

[B36] Hartley D. E. Elsabagh S. File S. E. (2004). Binge drinking and sex: effects on mood and cognitive function in healthy young volunteers. *Pharmacol. Biochem. Behav.* 78 611–619. 10.1016/j.pbb.2004.04.027 15251270

[B37] Heffernan T. Clark R. Bartholomew J. Ling J. Stephens S. (2010). Does binge drinking in teenagers affect their everyday prospective memory? *Drug Alcohol Depend.* 109 73–78. 10.1016/j.drugalcdep.2009.12.013 20071106

[B38] Hill B. Elliott E. Shelton J. Pella R. O’Jile J. Gouvier W. (2010). Can we improve the clinical assessment of working memory? An evaluation of the WAIS-III using a Working Memory criterion construct. *J. Clin. Exp. Neuropsychol.* 32 315–323. 10.1080/13803390903032529 19657913 PMC2854874

[B39] Jacobus J. Tapert S. F. (2013). Neurotoxic effects of alcohol in adolescence. *Annu. Rev. Clin. Psychol.* 9 703–721. 10.1146/annurev-clinpsy-050212-185610 23245341 PMC3873326

[B40] Jennison K. M. (2004). The short-term effects and unintended long-term consequences of binge drinking in college: a 10-year follow-up study. *Am. J. Drug Alcohol Abuse* 30 659–684. 10.1081/ADA-200032331 15540499

[B41] Johnson C. A. Xiao L. Palmer P. Sun P. Wang Q. Wei Y. (2008). Affective decision-making deficits, linked to adysfunctional ventromedial prefrontal cortex, revealed in 10th grade Chineseadolescent binge drinkers. *Neuropsychologia* 46 714–726. 10.1016/j.neuropsychologia.2007.09.012 17996909 PMC3498846

[B42] Jones S. A. Cservenka A. Nagel B. J. (2016). Binge drinking impacts dorsal striatal response during decision making in adolescents. *Neuroimage* 129 378–388. 10.1016/j.neuroimage.2016.01.044 26826511 PMC4803619

[B43] Jurado M. B. Rosselli M. (2007). The elusive nature of executive functions: a review of our current understanding. *Neuropsychol. Rev.* 17 213–233. 10.1007/s11065-007-9040-z 17786559

[B44] Kivimaki P. Kekkonen V. Valtonen H. Tolmunen T. Honkalampi K. Tacke U. (2014). Alcohol use among adolescents, aggressive behaviour, and internalizing problems. *J. Adolesc.* 37 945–951. 10.1016/j.adolescence.2014.06.011 25038493

[B45] Kono T. Matsuo K. Tsunashima K. Kasai K. Takizawa R. Rogers M. A. (2007). Multiple-time replicability of near-infrared spectroscopy recording during prefrontal activation task in healthy men. *Neurosci. Res.* 57 504–512. 10.1016/j.neures.2006.12.007 17250915

[B46] Lacaille H. Duterte-Boucher D. Liot D. Vaudry H. Naassila M. Vaudry D. (2015). Comparison of the deleterious effects of binge drinking-like alcohol exposure in adolescent and adult mice. *J. Neurochem.* 132 629–641. 10.1111/jnc.13020 25556946

[B47] Landa N. Castillo A. Fernández Montalvo J. López J. J. Lorea I. Tirapu J. (2006). Alteraciones neuropsicológicas en alcohólicos: un estudio exploratorio. *Adicciones* 18 49–59. 10.1080/13803390903032529

[B48] Lezak M. D. Howieson D. B. Loring D. W. Hannay H. J. Fischer J. S. (2004). *Neuropsychological Assessment*, 4th Edn New York, NY: Oxford University Press.

[B49] López-Caneda E. Cadaveira F. Crego A. Doallo S. Corral M. Gómez-Suárez A. (2013). Effects of a persistent binge drinking pattern of alcohol consumption in young people: a follow-up study using event-related potentials. *Alcohol Alcohol.* 48 464–471. 10.1093/alcalc/agt046 23695975

[B50] López-Caneda E. Cadaveira F. Crego A. Gómez-Suárez A. Corral M. Parada M. (2012). Hyperactivation of right inferior frontal cortex in young binge drinkers during response inhibition: a follow-up study. *Addiction* 107 1796–1808. 10.1111/j.1360-0443.2012.03908.x 22487028

[B51] López-Caneda E. Rodríguez Holguín S. Corral M. Doallo S. Cadaveira F. (2014). Evolution of the binge drinking pattern in college students: neurophysiological correlates. *Alcohol* 48 407–418. 10.1016/j.alcohol.2014.01.009 24835220

[B52] Luna B. Padmanabhan A. O’Hearn K. (2010). What has fMRI told us about the development of cognitive control through adolescence? *Brain Cogn.* 72 101–113. 10.1016/j.bandc.2009.08.005 19765880 PMC2815087

[B53] Martínez M. V. Manoiloff L. M. V. (2010). Evaluación neuropsicológica de la función ejecutiva en adolescentes con diferentes patrones de consumo de alcohol. *Rev. Argent. Cienc. Comport.* 2 14–23.

[B54] McCarthy D. M. Niculete M. E. Treloar H. R. Morris D. H. Bartholow B. D. (2012). Acute alcohol effects on impulsivity: associations with drinking and driving behavior. *Addiction* 107 2109–2114. 10.1111/j.1360-0443.2012.03974.x 22690907 PMC3449018

[B55] Miller J. W. Naimi T. S. Brewer R. D. Jones S. E. (2007). Binge drinking and associated health risk behaviors among high school students. *Pediatrics* 119 76–85. 10.1542/peds.2006-1517 17200273

[B56] Mitrushina M. Boone K. B. Razani J. D’Elia L. F. (2005). *Handbook of Normative Data for Neuropsychological Assessment*, 2nd Edn New York, NY: Oxford University Press.

[B57] Mota N. Parada M. Crego A. Doallo S. Caamaño-Isorna F. Rodríguez Holguín S. (2013). Binge drinking trajectory and neuropsychological functioning among university students: a longitudinal study. *Drug Alcohol Depend.* 133 108–114. 10.1016/j.drugalcdep.2013.05.024 23791027

[B58] Moure-Rodríguez L. Caamaño-Isorna F. Doallo S. Juan-Salvadores P. Corral M. Rodríguez-Holguín S. (2014). Heavy drinking and alcohol-related injuries in college students. *Gac. Sanit.* 28 376–380. 10.1016/j.gaceta.2014.02.017 24725631

[B59] Nelson T. F. Xuan Z. Lee H. Weitzman E. R. Wechsler H. (2009). Persistence of heavy drinking and ensuing consequences at heavy drinking colleges. *J. Stud. Alcohol Drugs* 70 726–734. 10.15288/jsad.2009.70.726 19737497

[B60] Oscar-Berman M. Marinkovic K. (2007). Alcohol: effects on neurobehavioral functions and the brain. *Neuropsychol. Rev.* 17 239–257. 10.1007/s11065-007-9038-6 17874302 PMC4040959

[B61] Parada M. Corral M. Caamaño-Isorna F. Mota N. Crego A. Rodríguez Holguín S. (2011a). Binge drinking and declarative memory in university students. *Alcohol. Clin. Exp. Res.* 35 1–10. 10.1111/j.1530-0277.2011.01484.x 21575014

[B62] Parada M. Corral M. Caamaño-Isorna F. Mota N. Crego A. Rodríguez-Holguín S. (2011b). Definition of adolescent binge drinking. *Adicciones* 23 53–63. 10.20882/adicciones.167 21503564

[B63] Parada M. Corral M. Mota N. Crego A. Rodríguez Holguín S. Cadaveira F. (2012). Executive functioning and alcohol binge drinking in university students. *Addict. Behav.* 37 167–172. 10.1016/j.addbeh.2011.09.015 21996093

[B64] Peele S. Brodsky A. (2000). Exploring psychological benefits associated with moderate alcohol use: a necessary corrective to assessments of drinking outcomes? *Drug Alcohol Depend.* 60 221–247. 10.1016/S0376-8716(00)00112-5 11053757

[B65] Petit G. Maurage P. Kornreich C. Verbanck P. Campanella S. (2014). Binge drinking in adolescents: a review of neurophysiological and neuroimaging research. *Alcohol Alcohol.* 49 198–206. 10.1093/alcalc/agt172 24302160

[B66] Pleil K. E. Lowery-Gionta E. G. Crowley N. A. Li C. Marcinkiewcz C. A. Rose J. H. (2015). Effects of chronic ethanol exposure on neuronal function in the prefrontal cortex and extended amygdala. *Neuropharmacology* 99 735–749. 10.1016/j.neuropharm.2015.06.017 26188147 PMC4781662

[B67] Popovici I. French M. T. (2013). Binge drinking and sleep problems among young adults. *Drug Alcohol Depend.* 132 207–215. 10.1016/j.drugalcdep.2013.02.001 23466223 PMC3748176

[B68] Randall D. C. Elsabagh S. M. Hartley D. E. File S. E. (2004). Does drinking have effects on mood and cognition in male and female students? *Pharmacol. Biochem. Behav.* 78 629–638. 10.1016/j.pbb.2004.04.029 15251272

[B69] Reitan R. M. (1992). *Trail Making Test: Manual for an Administration and Scoring.* Tucson, AZ: Reitan Neuropsychology Laboratory.

[B70] Richardson J. T. (2007). Measures of short-term memory: a historical review. *Cortex* 43 635–650. 10.1016/S0010-9452(08)70493-3 17715798

[B71] Ridderinkhof K. R. Ullsperger M. Crone E. A. Nieuwenhuis S. (2004). The role of the medial frontal cortex in cognitive control. *Science* 306 443–447. 10.1126/science.1100301 15486290

[B72] Rubia K. (2013). Functional brain imaging across development. *Eur. Child Adolesc Psychiatry* 22 719–731. 10.1007/s00787-012-0291-8 22729957 PMC3853580

[B73] Rubia K. Overmeyer S. Taylor E. Brammer M. Williams S. C. Simmons A. (2000). Functional frontalisation with age: mapping neurodevelopmental trajectories with fMRI. *Neurosci. Biobehav. Rev.* 24 13–19. 10.1016/S0149-7634(99)00055-x 10654655

[B74] Rueda M. R. Fan J. McCandliss B. D. Halparin J. D. Gruber D. B. Lercari L. P. (2004). Development of attentional networks in childhood. *Neuropsychologia* 42 1029–1040. 10.1016/j.neuropsychologia.2003.12.012 15093142

[B75] Sanhueza C. García-Moreno L. M. Expósito J. (2011). Weekend alcoholism in youth and neurocognitive aging. *Psicothema* 23 209–214. 21504671

[B76] Saunders J. B. Aasland O. G. Babor T. F. de la Fuente J. R. Grant M. (1993). Development of the Alcohol Use Disorders Identification Test (AUDIT): WHO collaborative project on early detection of persons with harmful alcohol consumption-II. *Addiction* 88 791–804. 10.1111/j.1360-0443.1993.tb02093.x 8329970

[B77] Scaife J. C. Duka T. (2009). Behavioural measures of frontal lobe function in a population of young social drinkers with binge drinking pattern. *Pharmacol. Biochem. Behav.* 93 354–362. 10.1016/j.pbb.2009.05.015 19497334

[B78] Schweinsburg A. D. Schweinsburg B. C. Nagel B. J. Eyler L. T. Tapert S. F. (2011). Neural correlates of verbal learning in adolescent alcohol and marijuana users. *Addiction* 106 564–573. 10.1111/j.1360-0443.2010.03197.x 21134014 PMC3423457

[B79] Schweinsburg D. A. McQueeny T. Nagel B. J. Eylere L. T. Tapert S. F. (2010). A preliminary study of functional magnetic resonance imaging response during verbal encoding among adolescent binge drinkers. *Alcohol* 44 111–117. 10.1016/j.alcohol.2009.09.032 20113879 PMC2845466

[B80] Spagnolli F. Cerini R. Cardobi N. Barillari M. Manganotti P. Storti S. (2013). Brain modifications after acute alcohol consumption analyzed by resting state fMRI. *Magn. Reson. Imaging* 31 1325–1330. 10.1016/j.mri.2013.04.007 23680187

[B81] Spear L. (2014). Adolescents and alcohol: acute sensitivities, enhanced intake, and later consequences. *Neurotoxicol. Teratol.* 41 51–59. 10.1016/j.ntt.2013.11.006 24291291 PMC3943972

[B82] Squeglia L. M. Schweinsburg A. D. Pulido C. Tapert S. F. (2011). Adolescent binge drinking linked to abnormal spatial working memory brain activation: differential gender effects. *Alcohol. Clin. Exp. Res.* 35 1831–1841. 10.1111/j.1530-0277.2011.01527.x 21762178 PMC3183294

[B83] Stautz K. Cooper A. (2013). Impulsivity-related personality traits and adolescent alcohol use: a meta-analytic review. *Clin. Psychol. Rev.* 33 574–592. 10.1016/j.cpr.2013.03.003 23563081

[B84] Strauss E. Sherman E. M. S. Spreen O. (2006). *A Compendium of Neuropsychological Tests: Administration, Norms, and Commentary*, 3rd Edn New York, NY: Oxford University Press.

[B85] Stroop J. R. (1935). Studies of interference in series verbal reactions. *J. Exp. Psychol.* 18 643–662. 10.1037/h0054651

[B86] Tapert S. F. Brown S. A. (1999). Neuropsychological correlates of adolescent substance abuse: four-year outcomes. *J. Int. Neuropsychol. Soc.* 5 481–493. 10.1017/S1355617799566010 10561928

[B87] The European School Survey Project on Alcohol and Other Drugs [ESPAD] (2011). *The 2011 ESPAD Report. Substance Use Among Students in 36 European Countries.* Lisbon: The European School Survey Project on Alcohol and Other Drugs.

[B88] Townshend J. M. Duka T. (2005). Binge drinking, cognitive performance and mood in a population of young social drinkers. *Alcohol. Clin. Exp. Res.* 29 317–325. 10.1097/01.ALC.0000156453.05028.F5 15770105

[B89] Trantham-Davidson H. Chandler L. J. (2015). Alcohol-induced alterations in dopamine modulation of prefrontal activity. *Alcohol* 49 773–779. 10.1016/j.alcohol.2015.09.001 26558348 PMC4691370

[B90] Tupak S. V. Badewien M. Dresler T. Hahn T. Ernst L. H. Herrmann M. J. (2012). Differential prefrontal and frontotemporal oxygenation patterns during phonemic and semantic verbal fluency. *Neuropsychologia* 50 1565–1569. 10.1016/j.neuropsychologia.2012.03.009 22426205

[B91] Wechsler D. (1997). *Wechsler Memory Scale-III.* San Antonio. TX: The Psychological Corporation.

[B92] Weissenborn R. Duka T. (2003). Acute alcohol effects on cognitive function in social drinkers: their relationship to drinking habits. *Psychopharmacology* 165 306–312. 10.1007/s00213-002-1281-1 12439627

[B93] White A. Hingson R. (2014). The burden of alcohol use: excessive alcohol consumption and related consequences among college students. *Alcohol Res.* 35 201–218. 10.35946/arcr.v35.2.11 PMC3908712 24881329

[B94] World Health Organization (2000). *International Guide for Monitoring Alcohol Consumption And Related Harm.* Geneva: WHO.

[B95] Xiao L. Bechara A. Gong Q. Huang X. Li X. Xue G. (2013). Abnormal affective decision making revealed in adolescent binge drinkers using a functional magnetic resonance imaging study. *Psychol. Addict. Behav.* 27 443–454. 10.1037/a0027892 22486330

[B96] Yurgelun-Todd D. A. (2007). Emotional and cognitive changes during adolescence. *Curr. Opin. Neurobiol.* 17 251–257. 10.1016/j.conb.2007.03.009 17383865

[B97] Zeigler D. W. Wang C. C. Yoast R. A. Dickinson B. D. McCaffree M. A. Robinowitz C. B. (2005). The neurocognitive effects of alcohol on adolescents and college students. *Prev. Med.* 40 23–32. 10.1016/j.ypmed.2004.04.044 15530577

[B98] Zelazo P. D. Carlson S. M. Kesek A. (2008). “Development of executive function in childhood,” in *Handbook of Developmental Cognitive Neuroscience*, 2nd Edn, eds Nelson C. A. Luciana M. (Cambridge, MA: MIT Press), 553–574.

[B99] Zheng W. B. (2017). “Acute ethanol-induced changes in microstructural and metabolite concentrations on the brain: noninvasive functional brain imaging,” in *Addictive Substances and Neurological Disease. Alcohol, Tobacco, Caffeine, and Drugs of Abuse in Everyday Lifestyles*, eds Watson R. R. Zibadi S. (San Diego, CA: Academic Press), 3–9.

[B100] Zölliga J. Martina M. Kliegel M. (2010). Forming intentions successfully: differential compensational mechanisms of adolescents and old adults. *Cortex* 46 575–589. 10.1016/j.cortex.2009.09.010 19875110

